# *trans*-Dichloridobis(triphenyl­phosphine)palladium(II)^1^

**DOI:** 10.1107/S1600536808008337

**Published:** 2008-04-04

**Authors:** Josefina Pons, Jordi García-Antón, Xavier Solans, Mercè Font-Bardia, Josep Ros

**Affiliations:** a Departamento de Química, Unitat de Química Inorgànica, Universitat Autònoma de Barcelona, 08193 Bellaterra, Barcelona, Spain; b Departament de Cristallografia, Universitat de Barcelona, Martí i Franquès, sn, E-08028 Barcelona, Spain

## Abstract

The title compound, [PdCl_2_{P(C_6_H_5_)_3_}_2_], has a slightly distorted square-planar geometry, with the chloride ligands coordinated in a *trans* configuration. The Pd atom is located on a centre of inversion.

## Related literature

For related literature, see: Ferguson *et al.* (1982 ▶); Kitano *et al.* (1983 ▶); La Monica & Ardizzoia (1997 ▶); Montoya *et al.* (2005 ▶); Montoya *et al.* (2006 ▶); Mukherjee (2000 ▶); Oilunkaniemi *et al.* (2003 ▶); Stark *et al.* (1997 ▶); Steyl (2006 ▶); Trofimenko (1972 ▶, 1986 ▶).
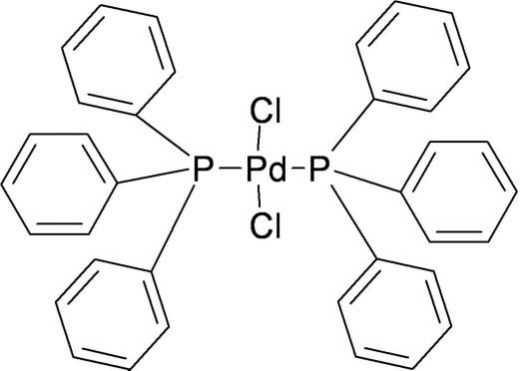


## Experimental

### 

#### Crystal data

[PdCl_2_(C_18_H_15_P)_2_]
*M* *_r_* = 701.84
Monoclinic, 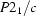

*a* = 9.296 (5) Å
*b* = 19.889 (8) Å
*c* = 10.621 (6) Å
β = 121.71 (4)°
*V* = 1670.6 (15) Å^3^
*Z* = 2
Mo *K*α radiation
μ = 0.83 mm^−1^
*T* = 293 (2) K
0.2 × 0.17 × 0.16 mm

#### Data collection

Mar Research MAR345 diffractometer with image-plate detector
Absorption correction: multi-scan (*SADABS*; Bruker, 1999 ▶) *T*_min_ = 0.85, *T*_max_ = 0.87
4898 measured reflections
4898 independent reflections
3143 reflections with *I* > 2σ(*I*)
*R*_int_ = 0.033

#### Refinement

*R*[*F* ^2^ > 2σ(*F* ^2^)] = 0.037
*wR*(*F* ^2^) = 0.077
*S* = 0.93
4898 reflections
187 parameters
7 restraints
H-atom parameters constrained
Δρ_max_ = 0.56 e Å^−3^
Δρ_min_ = −0.36 e Å^−3^

### 

Data collection: *MARXDS* (Kabsch, 1988 ▶); cell refinement: *AUTOMAR* (Kabsch, 1988 ▶); data reduction: *MARSCALE* (Kabsch, 1988 ▶); program(s) used to solve structure: *SHELXS97* (Sheldrick, 2008 ▶); program(s) used to refine structure: *SHELXL97* (Sheldrick, 2008 ▶); molecular graphics: *ORTEP-3* (Farrugia, 1997 ▶); software used to prepare material for publication: *PLATON* (Spek, 2003 ▶).

## Supplementary Material

Crystal structure: contains datablocks I, global. DOI: 10.1107/S1600536808008337/bt2685sup1.cif

Structure factors: contains datablocks I. DOI: 10.1107/S1600536808008337/bt2685Isup2.hkl

Additional supplementary materials:  crystallographic information; 3D view; checkCIF report

## Figures and Tables

**Table:** 

Pd—Cl	2.3111 (13)
Pd—P	2.3721 (10)

**Table:** 

Cl—Pd—P	87.62 (4)
Cl^i^—Pd—P	92.38 (4)

**Table:** 

Cl—Pd—P—C1	41.9 (2)
Cl—Pd—P—C13	−75.7 (2)
Cl—Pd—P—C7	163.9 (2)

Symmetry code: (i) 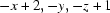.

**Fig. 1.**
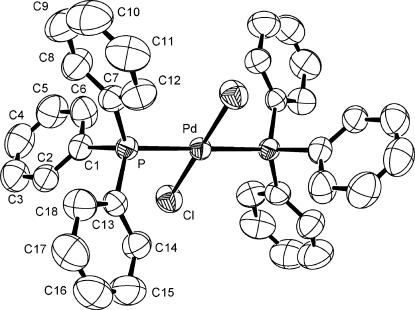
trans-Dichlorobis(triphenylphosphine)palladium(II)

## supplementary crystallographic information

### Comment

The coordination chemistry of pyrazole derived ligands has been extensively studied in recent years (Trofimenko, 1972, 1986; La Monica *et al.*, 1997; Mukherjee, 2000). Recently, in our laboratory the synthesis and characterization of a family of 1,3,5-pyrazole derived ligands have been developed (Montoya *et al. *2005) and we have studied the reactivity towards divalent metal ions. The reaction of [PdCl_2_*L*^1^] (*L*^1^ = 2-(1-ethyl-5-phenyl-1*H*-pyrazol-3-yl)pyridine with AgBF_4_ followed by the addition of PPh_3_ and NaBPh_4_ yields the compound [Pd(*L*^1^)(PPh_3_)_2_](BPh_4_)_2_ (Montoya *et al.*, 2006). The title compound was obtained when the triphenylphosphine ligand was added before the precipitation of the chloride ions with AgBF_4_. In this way, PPh_3_ ligands displace *L*^1^ to form *trans*-[PdCl_2_(PPh_3_)_2_] (1).

Related compounds are *trans*-[PdCl_2_(PPh_3_)_2_] (2) (Ferguson *et al.*, 1982), *trans*-[PdCl_2_(PPh_3_)_2_].C_6_H_4_Cl_2_ (Kitano *et al.*,1983), *trans*-[PdCl_2_(PPh_3_)_2_].2CHCl_3_ (Stark *et al*., 1997), *trans*-[PdCl_2_(PPh_3_)_2_].CH_2_Cl_2_ (Oilunkaniemi *et al.*, 2003), and *trans*-[PdCl_2_(PPh_3_)_2_].C_2_H_4_Cl_2_ (Steyl, 2006). There are no solvent molecules present in the structure described in this paper. The same behaviour was found for the structure described by Ferguson (2), but differences have been found in the crystal systems and space groups [triclinic P1 (2); monoclinic *P*2_1_/*c*, (1)]. Moreover, the Pd—Cl and Pd—P bond distances (2.3111 (13) Å and 2.3721 (10) Å, respectively) in complex (1) are slightly longer than those found in complex (2) (2.290 (1) Å and 2.337 (1) Å, respectively).

### Experimental

Treatment of 0.14 mmol (0.060 g) of [PdCl_2_(*L*^1^)] (*L*^1^ = 2-(1-ethyl-5-phenyl-1*H*-pyrazol-3-yl)pyridine) with 0.28 mmol (0.054 g) of PPh_3_ in 10 ml of dichloromethane and 10 ml of methanol provokes the displacement of the pyrazolic ligand from the coordinative sphere of the metallic cation and the formation of *trans*-[PdCl_2_(PPh_3_)_2_]. This complex precipitates as a yellow solid and was filtered and dried under vacuum. Single crystals were obtained by recrystallization of the complex in dichloromethane/diethyl ether 1:1. Yield: 0.080 g (81%) - C_36_H_30_Cl_2_P_2_Pd (701.84). % C, 61.60; H, 4.30; found: C, 61.33; H, 4.42;. IR (KBr, cm^-1^): ν (C—H)_ar_ 3047; δ (C—H)_ar_ 1437; δ (C—H)_oop_ 693. IR (polyethylene, cm^-1^): ν 376, 358 (Pd—P), ν (Pd—Cl). ^1^H NMR (250 MHz, [D_1_]-chloroform solution) δ = 7.71 (m, 2H, PPh_3_*ortho*), 7.44–7.35 (m, 3H, PPh_3_). ^13^C{^1^H} NMR (63 MHz, [D_1_]-chloroform solution) δ = 135.5, 131.0, 130.0, 128.5 (PPh_3_). ^31^P{^1^H} NMR (81 MHz, [D_1_]-chloroform solution) δ = -21.1 (s, PPh_3_).

### Refinement

We had serious problems growing up good crystals of reasonable size and quality and, in all cases, we obtained twinned crystals with very broad reflections (bad mosaic structure). Measurement were done in a image plate difractometer which only measure in a single /f angle.

All H atoms were computed and refined, using a riding model, with an isotropic temperature factor equal to 1.2 times the equivalent temperature factor of the atom which are bonded.

### Crystal data

**Table:**

[PdCl_2_(C_18_H_15_P_1_)_2_]	*F*_000_ = 712
*M**_r_* = 701.84	*D*_x_ = 1.395 Mg m^−^^3^
Monoclinic, *P*2_1_/*c*	Mo *K*α radiation radiation λ = 0.71073 Å
*a* = 9.296 (5) Å	Cell parameters from 26 reflections
*b* = 19.889 (8) Å	θ = 3–31º
*c* = 10.621 (6) Å	µ = 0.83 mm^−^^1^
β = 121.71 (4)º	*T* = 293 (2) K
*V* = 1670.6 (15) Å^3^	Prism, yellow
*Z* = 2	0.2 × 0.17 × 0.16 mm

### Data collection

**Table:**

MAR345 with image-plate detector diffractometer	4898 independent reflections
Radiation source: fine-focus sealed tube	3143 reflections with *I* > 2σ(*I*)
Monochromator: graphite	*R*_int_ = 0.033
*T* = 293(2) K	θ_max_ = 33.3º
φ scans	θ_min_ = 3.8º
Absorption correction: multi-scan(SADABS; Bruker, 1999)	*h* = −14→12
*T*_min_ = 0.85, *T*_max_ = 0.87	*k* = 0→30
4898 measured reflections	*l* = 0→16

### Refinement

**Table:**

Refinement on *F*^2^	Secondary atom site location: difference Fourier map
Least-squares matrix: full	Hydrogen site location: inferred from neighbouring sites
*R*[*F*^2^ > 2σ(*F*^2^)] = 0.037	H-atom parameters constrained
*wR*(*F*^2^) = 0.077	*w* = 1/[σ^2^(*F*_o_^2^) + (0.0269*P*)^2^] where *P* = (*F*_o_^2^ + 2*F*_c_^2^)/3
*S* = 0.93	(Δ/σ)_max_ = 0.002
4898 reflections	Δρ_max_ = 0.56 e Å^−^^3^
187 parameters	Δρ_min_ = −0.36 e Å^−^^3^
7 restraints	Extinction correction: none
Primary atom site location: structure-invariant direct methods	

### Special details

**Table:**

Geometry. All e.s.d.'s (except the e.s.d. in the dihedral angle between two l.s. planes) are estimated using the full covariance matrix. The cell e.s.d.'s are taken into account individually in the estimation of e.s.d.'s in distances, angles and torsion angles; correlations between e.s.d.'s in cell parameters are only used when they are defined by crystal symmetry. An approximate (isotropic) treatment of cell e.s.d.'s is used for estimating e.s.d.'s involving l.s. planes.
Refinement. Refinement of *F*^2^ against ALL reflections. The weighted *R*-factor *wR* and goodness of fit *S* are based on *F*^2^, conventional *R*-factors *R* are based on *F*, with *F* set to zero for negative *F*^2^. The threshold expression of *F*^2^ > σ(*F*^2^) is used only for calculating *R*-factors(gt) *etc*. and is not relevant to the choice of reflections for refinement. *R*-factors based on *F*^2^ are statistically about twice as large as those based on *F*, and *R*- factors based on ALL data will be even larger.

### Fractional atomic coordinates and isotropic or equivalent isotropic displacement parameters (Å^2^)

**Table:**

	*x*	*y*	*z*	*U*_iso_*/*U*_eq_	
Pd	1.0000	0.0000	0.5000	0.04342 (7)	
P	0.84764 (7)	0.09596 (3)	0.36120 (6)	0.04430 (13)	
Cl	0.97931 (10)	0.04208 (4)	0.69271 (7)	0.06833 (18)	
C1	0.6712 (3)	0.11530 (14)	0.3892 (3)	0.0580 (6)	
C2	0.6470 (3)	0.17570 (17)	0.4357 (3)	0.0657 (7)	
H2	0.7217	0.2106	0.4529	0.079*	
C3	0.5150 (5)	0.1869 (2)	0.4584 (4)	0.0889 (10)	
H3	0.5026	0.2279	0.4939	0.107*	
C4	0.4034 (5)	0.1351 (2)	0.4262 (4)	0.0969 (11)	
H4	0.3102	0.1429	0.4346	0.116*	
C5	0.4210 (5)	0.0737 (2)	0.3832 (4)	0.0976 (11)	
H5	0.3458	0.0394	0.3685	0.117*	
C6	0.5576 (4)	0.06221 (17)	0.3603 (4)	0.0811 (8)	
H6	0.5711	0.0207	0.3273	0.097*	
C7	0.7530 (3)	0.09435 (13)	0.1606 (3)	0.0614 (6)	
C8	0.5790 (4)	0.09538 (17)	0.0615 (3)	0.0835 (9)	
H8	0.5064	0.0960	0.0970	0.100*	
C9	0.5128 (6)	0.0955 (2)	−0.0899 (4)	0.1180 (16)	
H9	0.3962	0.0965	−0.1547	0.142*	
C10	0.6163 (7)	0.0942 (2)	−0.1452 (4)	0.1208 (16)	
H10	0.5706	0.0937	−0.2469	0.145*	
C11	0.7918 (6)	0.0935 (2)	−0.0474 (4)	0.0996 (12)	
H11	0.8631	0.0930	−0.0842	0.120*	
C12	0.8601 (4)	0.09361 (17)	0.1056 (3)	0.0774 (8)	
H12	0.9767	0.0932	0.1705	0.093*	
C13	0.9779 (3)	0.17318 (11)	0.4177 (2)	0.0491 (5)	
C14	1.1113 (3)	0.18023 (15)	0.5592 (3)	0.0659 (7)	
H14	1.1381	0.1455	0.6266	0.079*	
C15	1.2095 (5)	0.2397 (2)	0.6048 (4)	0.0953 (11)	
H15	1.3024	0.2438	0.7007	0.114*	
C16	1.1653 (5)	0.2915 (2)	0.5048 (5)	0.0961 (11)	
H16	1.2282	0.3310	0.5349	0.115*	
C17	1.0406 (5)	0.28661 (18)	0.3722 (5)	0.0945 (11)	
H17	1.0167	0.3220	0.3067	0.113*	
C18	0.9362 (4)	0.22755 (15)	0.3218 (3)	0.0761 (8)	
H18	0.8419	0.2256	0.2262	0.091*	

### Atomic displacement parameters (Å^2^)

**Table:**

	*U*^11^	*U*^22^	*U*^33^	*U*^12^	*U*^13^	*U*^23^
Pd	0.04515 (12)	0.04507 (12)	0.04158 (11)	0.00132 (11)	0.02386 (9)	0.00015 (10)
P	0.0441 (3)	0.0449 (3)	0.0430 (3)	0.0013 (2)	0.0222 (2)	0.0019 (2)
Cl	0.0810 (4)	0.0703 (4)	0.0607 (3)	0.0072 (3)	0.0421 (3)	−0.0001 (3)
C1	0.0524 (12)	0.0642 (15)	0.0558 (12)	0.0073 (9)	0.0274 (11)	0.0050 (10)
C2	0.0627 (16)	0.0724 (19)	0.0595 (15)	0.0074 (13)	0.0304 (13)	0.0001 (11)
C3	0.088 (2)	0.104 (3)	0.081 (2)	0.018 (2)	0.0492 (18)	−0.0020 (18)
C4	0.081 (2)	0.130 (4)	0.092 (2)	0.020 (2)	0.0533 (19)	0.014 (2)
C5	0.075 (2)	0.108 (3)	0.115 (3)	−0.009 (2)	0.054 (2)	0.007 (2)
C6	0.0719 (18)	0.0721 (19)	0.111 (2)	0.0005 (13)	0.0560 (18)	0.0040 (18)
C7	0.0689 (12)	0.0598 (15)	0.0481 (11)	0.0004 (12)	0.0256 (9)	0.0019 (10)
C8	0.0704 (13)	0.094 (2)	0.0730 (14)	0.0068 (17)	0.0284 (13)	0.0058 (16)
C9	0.111 (3)	0.130 (3)	0.0689 (15)	−0.001 (3)	0.016 (2)	0.002 (2)
C10	0.148 (4)	0.126 (4)	0.070 (2)	−0.017 (3)	0.045 (3)	−0.003 (2)
C11	0.129 (3)	0.111 (3)	0.082 (2)	−0.022 (3)	0.071 (2)	−0.007 (2)
C12	0.0803 (19)	0.091 (2)	0.0613 (15)	−0.0088 (17)	0.0374 (14)	0.0021 (14)
C13	0.0493 (11)	0.0486 (12)	0.0566 (10)	0.0000 (9)	0.0328 (9)	−0.0006 (9)
C14	0.0632 (15)	0.0688 (16)	0.0605 (11)	−0.0008 (12)	0.0290 (10)	−0.0002 (11)
C15	0.088 (2)	0.090 (2)	0.100 (2)	−0.0232 (18)	0.044 (2)	−0.020 (2)
C16	0.103 (3)	0.083 (2)	0.122 (3)	−0.024 (2)	0.073 (3)	−0.019 (2)
C17	0.117 (3)	0.071 (2)	0.105 (3)	−0.008 (2)	0.065 (2)	0.0084 (19)
C18	0.088 (2)	0.0688 (19)	0.0718 (17)	−0.0061 (16)	0.0424 (16)	0.0055 (13)

### Geometric parameters (Å, °)

**Table:**

Pd—Cl	2.3111 (13)	C8—C9	1.387 (5)
Pd—Cl^i^	2.3111 (13)	C8—H8	0.9300
Pd—P	2.3721 (10)	C9—C10	1.366 (6)
Pd—P^i^	2.3721 (10)	C9—H9	0.9300
P—C7	1.829 (3)	C10—C11	1.400 (6)
P—C13	1.849 (2)	C10—H10	0.9300
P—C1	1.855 (3)	C11—C12	1.400 (4)
C1—C2	1.361 (4)	C11—H11	0.9300
C1—C6	1.408 (4)	C12—H12	0.9300
C2—C3	1.387 (4)	C13—C14	1.362 (3)
C2—H2	0.9300	C13—C18	1.394 (4)
C3—C4	1.373 (5)	C14—C15	1.416 (4)
C3—H3	0.9300	C14—H14	0.9300
C4—C5	1.342 (5)	C15—C16	1.378 (5)
C4—H4	0.9300	C15—H15	0.9300
C5—C6	1.432 (5)	C16—C17	1.273 (6)
C5—H5	0.9300	C16—H16	0.9300
C6—H6	0.9300	C17—C18	1.436 (5)
C7—C8	1.391 (4)	C17—H17	0.9300
C7—C12	1.394 (4)	C18—H18	0.9300
			
Cl—Pd—Cl^i^	180.0	C9—C8—C7	120.5 (4)
Cl—Pd—P	87.62 (4)	C9—C8—H8	119.8
Cl^i^—Pd—P	92.38 (4)	C7—C8—H8	119.8
Cl—Pd—P^i^	92.38 (4)	C10—C9—C8	121.0 (4)
Cl^i^—Pd—P^i^	87.62 (4)	C10—C9—H9	119.5
P—Pd—P^i^	180.0	C8—C9—H9	119.5
C7—P—C13	102.99 (12)	C9—C10—C11	119.4 (4)
C7—P—C1	105.43 (13)	C9—C10—H10	120.3
C13—P—C1	105.00 (12)	C11—C10—H10	120.3
C7—P—Pd	118.32 (9)	C10—C11—C12	120.2 (4)
C13—P—Pd	113.03 (8)	C10—C11—H11	119.9
C1—P—Pd	110.89 (9)	C12—C11—H11	119.9
C2—C1—C6	119.6 (3)	C7—C12—C11	119.9 (3)
C2—C1—P	124.7 (2)	C7—C12—H12	120.1
C6—C1—P	115.7 (2)	C11—C12—H12	120.1
C1—C2—C3	122.3 (3)	C14—C13—C18	117.9 (2)
C1—C2—H2	118.8	C14—C13—P	120.31 (19)
C3—C2—H2	118.8	C18—C13—P	121.59 (19)
C4—C3—C2	117.2 (4)	C13—C14—C15	120.9 (3)
C4—C3—H3	121.4	C13—C14—H14	119.6
C2—C3—H3	121.4	C15—C14—H14	119.6
C5—C4—C3	123.8 (4)	C16—C15—C14	118.9 (3)
C5—C4—H4	118.1	C16—C15—H15	120.6
C3—C4—H4	118.1	C14—C15—H15	120.6
C4—C5—C6	118.7 (4)	C17—C16—C15	121.7 (4)
C4—C5—H5	120.6	C17—C16—H16	119.2
C6—C5—H5	120.6	C15—C16—H16	119.2
C1—C6—C5	118.2 (3)	C16—C17—C18	121.3 (3)
C1—C6—H6	120.9	C16—C17—H17	119.4
C5—C6—H6	120.9	C18—C17—H17	119.4
C8—C7—C12	119.1 (3)	C13—C18—C17	119.2 (3)
C8—C7—P	122.5 (3)	C13—C18—H18	120.4
C12—C7—P	118.4 (2)	C17—C18—H18	120.4
			
Cl—Pd—P—C1	41.9 (2)	Cl—Pd—P—C7	163.9 (2)
Cl—Pd—P—C13	−75.7 (2)		

Symmetry codes: (i) −*x*+2, −*y*, −*z*+1.
